# GLP-1 receptor agonists beyond glycemic control: integrated cardio-renal-metabolic protection across the cardiovascular kidney metabolic continuum

**DOI:** 10.1186/s40842-026-00322-3

**Published:** 2026-09-07

**Authors:** Ahmed Elsayed, Wadah Jason Ayoub, Norhan Elsayed, Joshua Stephens, Ndausung Udongwo, Yousif Elsayed, Jalal K. Ghali

**Affiliations:** 1 Department of Gastroenterology and Hepatology, Mayo Clinic, Rochester, MN 55905 USA; 2 Faculty of Medicine, Alexandria University, Alexandria, 5372066 Egypt; 3 Department of Nephrology, Medical College of Wisconsin, Milwaukee, WI 53226 USA; 4 Department of Cardiology, Morehouse School of Medicine, Atlanta, GA 30310 USA; 5 Department of Internal Medicine, Faculty of Medicine, Alexandria University, Alexandria, Egypt

**Keywords:** GLP-1 receptor agonists, Cardiovascular-renal-metabolic syndrome, Major adverse cardiovascular events, Chronic kidney disease, Heart failure, Cardiovascular outcomes trials

## Abstract

Glucagon-like peptide-1 receptor agonists (GLP-1RAs) have undergone a fundamental repositioning, once defined by their glycemic effects, they are now established as multi-organ protective agents operating across the cardiovascular-kidney-metabolic (CKM) continuum with benefits that extend substantially beyond glucose lowering, though the precise contribution of glycemic versus non-glycemic mechanisms varies by outcome and population. Major cardiovascular and renal outcome trials; LEADER, REWIND, AMPLITUDE-O, SELECT, FLOW, and SOUL, collectively demonstrate 13–20% reductions in major adverse cardiovascular events (MACE) and meaningful attenuation of chronic kidney disease (CKD) progression in both diabetic and non-diabetic populations. Exploratory mediation analyses from SELECT suggest that a substantial fraction of cardiovascular benefit is not explained by measured cardiometabolic risk factor changes, a finding that is hypothesis-generating and informs ongoing mechanistic investigation. This narrative review synthesizes mechanistic and clinical evidence for integrated GLP-1RA CKM protection, evaluates combination strategies with Sodium-Glucose Cotransporter 2 (SGLT2) inhibitors, and confronts the implementation gap that separates extraordinary trial efficacy from modest real-world uptake, with rigorous distinction between RCT-level evidence, observational findings, and mechanistic hypotheses. We propose a CKM Disease-Modification Framework as a conceptual scaffold for clinical decision-making, rather than treating cardiovascular, renal, and metabolic risk as separate targets requiring sequential intervention. This framework positions GLP-1RAs as upstream disease-modifying agents whose early deployment across the continuum, guided by dominant comorbidity rather than glycemic status, offers the greatest opportunity for organ protection. Taken together, the evidence positions GLP-1RAs not as adjuncts to standard cardiometabolic care, but as a foundational pillar of it, one whose population-level impact is currently constrained not by biology but by cost, access, and awareness.

**Graphical Abstract:**

**Figure:**
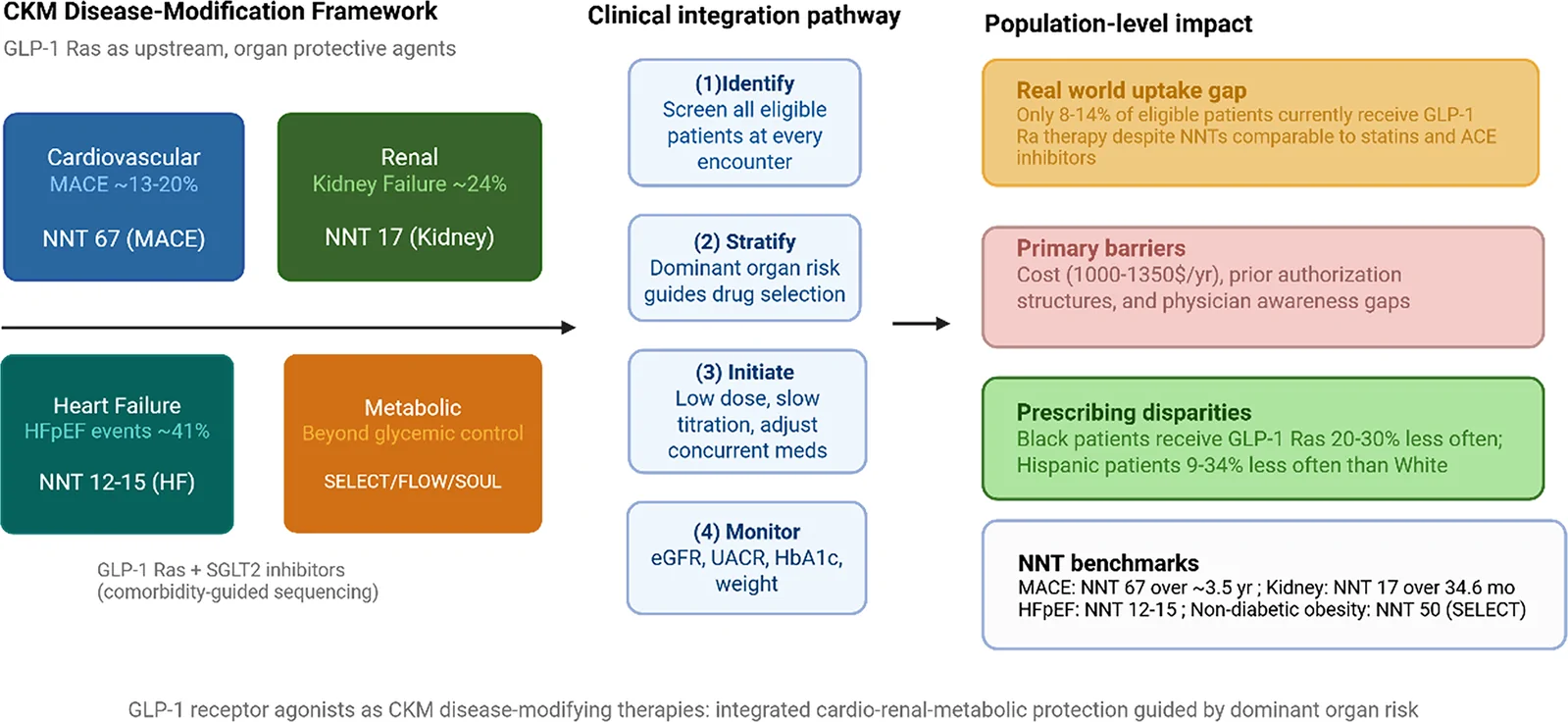

## Research insights

What is currently known about this topic?

What is the key research question?

What is new?

How might this study influence clinical practice?

GLP-1RAs reduce MACE by 13–20% across major cardiovascular outcomes trials. SELECT established cardio-renal benefits beyond the diabetic population; FLOW demonstrated dedicated renal outcomes in patients with type 2 diabetes and CKD. Real-world uptake remains 8-14% of eligible patients despite strong trial evidence.

Can GLP-1 receptor agonists be repositioned as disease-modifying therapies across the full CKM continuum, independent of glycemic control?

We propose the CKM Disease-Modification Framework as a novel clinical decision model. GLP-1RA benefits exceed what measured risk factor changes alone can explain. A comorbidity-guided, 4-step integration pathway is proposed for clinical practice.

Recognizing GLP-1RAs as CKM disease-modifying agents may shift prescribing from glucose-centric to organ-protective, earlier intervention across all eligible populations.

## Introduction

Type 2 diabetes, obesity, cardiovascular disease, and CKD converge across the CKM continuum, a framework in which insulin resistance, inflammation, and hemodynamic dysfunction drive progressive multi-organ damage [1]. Despite guideline-directed renin-angiotensin-aldosterone system (RAAS) blockade and statin therapy, substantial residual cardiovascular and renal risk persists [2].

Two drug classes have redefined this landscape: SGLT2 inhibitors and GLP-1 receptor agonists. While SGLT2 inhibitors reduce kidney disease progression and heart failure hospitalization predominantly through hemodynamic mechanisms, GLP-1RAs act as multi-organ protectors operating substantially independent of glucose lowering [3]. Two trials have been particularly defining. The SELECT trial (2023) demonstrated a 20% reduction in MACE among 17,604 non-diabetic patients with obesity and established atherosclerotic cardiovascular disease (ASCVD), establishing GLP-1RA benefit beyond the diabetic population [4]. The FLOW trial (2024) established GLP-1RA efficacy specifically in diabetic kidney disease, reducing the risk of kidney failure by 27% over a median of 34.6 months [5]. Taken together, these trials signal a paradigm shift; GLP-1RAs are no longer best understood solely as glucose-lowering drugs, but as cardio-renal protective agents whose benefits in high-risk populations appear to operate through mechanisms that are not fully explained by glycemic control alone, though the relative contribution of glycemic versus non-glycemic pathways remains an active area of investigation.

Exploratory mediation analyses from SELECT suggest that only approximately 30–40% of the MACE reduction attributable to semaglutide is explained by changes in measured cardiometabolic risk factors, with the remainder not accounted for by the variables measured [6]. This observation must be interpreted with substantial caution; the analysis was exploratory and not pre-specified as a primary endpoint, mediation models assume no unmeasured confounding between mediators and the outcome, and the residual unexplained fraction may reflect measurement limitations or unmeasured confounders rather than distinct pleiotropic mechanisms. The finding is hypothesis-generating and does not establish specific causal pathways; it is included here because it informs the rationale for ongoing mechanistic research, not because it constitutes evidence of a defined biological mechanism.

Despite this evidence base, real-world adoption of GLP-1RAs remains limited to approximately 8–14% of eligible patients, driven by cost, insurance restrictions, and limited physician recognition of cardio-renal benefits independent of glycemic targets [7]. This review synthesizes evidence to position GLP-1RAs as cardio-renal protective agents, evaluate combination strategies with SGLT2 inhibitors, and define the implementation and research priorities needed to bridge efficacy and practice. To structure this synthesis, we propose the CKM Disease-Modification Framework; a clinical decision model that repositions GLP-1RAs as upstream disease-modifying agents across the cardiovascular-kidney-metabolic continuum, guided by dominant comorbidity rather than glycemic threshold, and deployed early rather than as rescue therapy after organ damage is established. Collectively, the evidence supports this repositioning as both scientifically justified and clinically urgent.

## Methods

### Study design

**Fig. 1:**
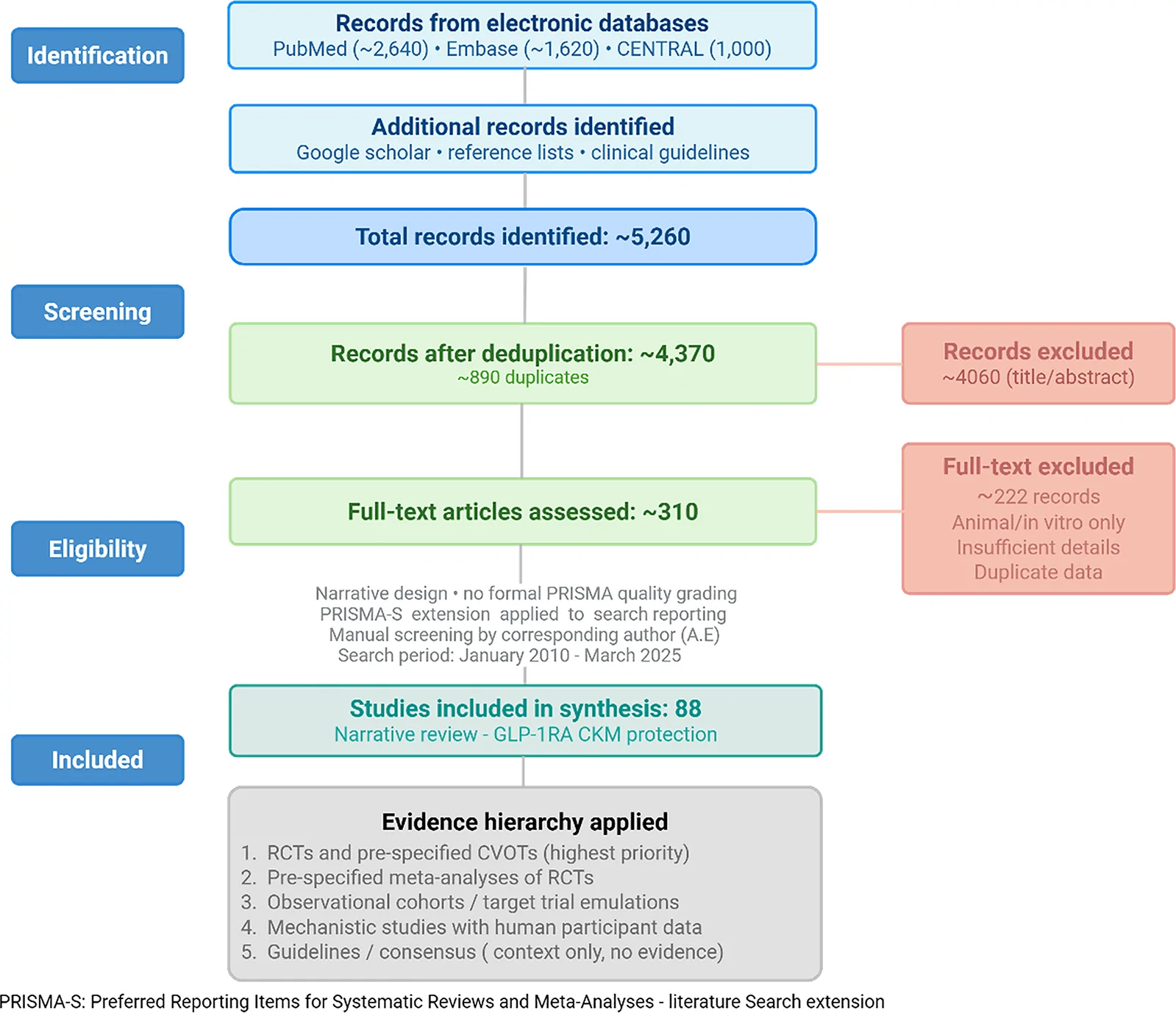
PRISMA-S flow diagram illustrating the literature search and study selection process. PRISMA-S = Preferred Reporting Items for Systematic Reviews and Meta-Analyses - literature Search extension

This narrative review synthesizes mechanistic and clinical evidence for GLP-1RA CKM protection. A narrative design was chosen to integrate evidence across biological mechanisms, cardiovascular and renal outcomes trials, combination therapy strategies, and implementation science. Although a narrative review does not require systematic search documentation, the search strategy reported here adheres to the PRISMA-S (Preferred Reporting Items for Systematic Reviews and Meta-Analyses - literature Search) extension to maximize search transparency [8] (Fig. 1). This approach carries inherent limitations: it is not pre-registered, does not employ formal study-level quality grading, and is subject to selection bias. Findings should be interpreted as an expert synthesis rather than a definitive systematic estimate of effect.

In plain terms, this review prioritized randomized controlled trials and pre-specified meta-analyses as the foundation for all clinical conclusions. Observational studies, target trial emulations, and post-hoc analyses are included to contextualize real-world applicability and generate hypotheses, but are explicitly labelled as such and not used to support efficacy claims. Mechanistic studies are included to explain biological plausibility, not to establish clinical benefit. This review did not attempt a full systematic review with study-level quality grading using tools such as GRADE or Cochrane risk-of-bias assessment; readers seeking that level of evidence appraisal should consult the pre-specified systematic reviews and meta-analyses cited throughout. The goal of this narrative synthesis is to integrate evidence across mechanism, outcomes, and implementation in a form that is clinically actionable with explicit signposting of where the evidence is strong, where it is preliminary, and where gaps require prospective investigation.

### Data sources and search strategy

A structured literature search was conducted across three electronic databases; PubMed, Embase, and the Cochrane Central Register of Controlled Trials (CENTRAL), for articles published from January 2010 through March 2025. Google Scholar was searched as a supplementary source to identify preprints, grey literature, and conference proceedings. The search timeframe of 2010 onward was chosen to capture the era of dedicated cardiovascular outcomes trials (CVOTs) for glucose-lowering therapies, initiated following the 2008 regulatory requirement for cardiovascular safety assessment.

Search terms were structured as combinations of MeSH headings and free-text keywords using the following Boolean strings:

String 1 (GLP-1RAs drug class): (“glucagon-like peptide-1 receptor agonist” OR “GLP-1RA” OR “GLP-1 receptor agonist” OR “semaglutide” OR “liraglutide” OR “dulaglutide” OR “exenatide” OR “albiglutide” OR “efpeglenatide” OR “tirzepatide”)

String 2 (cardiovascular and renal outcomes): (“cardiovascular outcomes” OR “major adverse cardiovascular events” OR “MACE” OR “cardiovascular death” OR “myocardial infarction” OR “stroke” OR “renal outcomes” OR “kidney failure” OR “eGFR decline” OR “albuminuria” OR “UACR”)

String 3 (disease domains): (“heart failure” OR “heart failure with preserved ejection fraction” OR “HFpEF” OR “chronic kidney disease” OR “diabetic kidney disease” OR “obesity” OR “type 2 diabetes” OR “cardiorenal metabolic syndrome” OR “cardiovascular-kidney-metabolic”)

Strings 1 through 3 were combined with AND. Study design filtering was not applied at the search stage; instead, study type was used as a criterion during the title/abstract and full-text screening phases, as described in section “Study selection and evidence prioritization”. This approach was adopted to maximize sensitivity of retrieval and avoid excluding relevant mechanistic or observational studies that would not have been captured by study design terms alone.

The combined database search retrieved approximately 5,260 records (PubMed: ~2,640; Embase: ~1,620; CENTRAL: ~1000). After removal of duplicates (~ 890 records), approximately 4,370 unique records were screened by title and abstract. Of these, approximately 310 full-text articles were assessed for eligibility against the inclusion criteria described in section “Study selection and evidence prioritization”. Following full-text review and application of the evidence hierarchy, 88 references were included in the final synthesis.

### Study selection and evidence prioritization

Study selection followed a hierarchical evidence-weighting framework rather than formal PRISMA screening, consistent with the narrative design of this review. Eligible study types, listed in descending order of evidentiary weight, were: (1) large randomized controlled trials, with pre-specified CVOTs given the highest priority; (2) pre-specified meta-analyses and systematic reviews of RCTs; (3) high-quality target trial emulations and propensity-matched observational cohorts with explicit confounding adjustment; and (4) mechanistic studies with direct human participant data, including biomarker substudy analyses from CVOTs and early-phase human pharmacodynamic studies. Articles published in English were included; non-English sources were excluded for feasibility. Single case reports were excluded. Studies were additionally excluded if they lacked sufficient methodological detail to assess internal validity.

A critical distinction was applied throughout the synthesis regarding mechanistic evidence. Mechanistic studies were eligible for inclusion only when they reported data from human participants, either as primary evidence (e.g., human endothelial cell culture studies with validated assays, early-phase human pharmacodynamic studies) or as biomarker sub-studies of completed RCTs. Animal and in vitro studies were included solely to provide biological plausibility context for mechanisms not yet confirmed in human data, and are explicitly labelled as preclinical throughout the text. No clinical conclusion in this review rests on preclinical evidence alone. Where a mechanistic pathway has been proposed in preclinical models but lacks human confirmation, this is stated explicitly in the relevant section. This distinction is applied consistently in section “Mechanisms of cardio-renal protection” (Mechanisms of Cardio-Renal Protection).

Where multiple publications from the same trial were available, including primary reports, pre-specified secondary analyses, and post-hoc subgroup analyses, the most comprehensive or most recent primary report was prioritized for core efficacy estimates. Pre-specified secondary analyses were cited for specific endpoints not addressed in the primary publication. Post-hoc analyses and exploratory sub-studies, including mediation analyses, are cited as hypothesis-generating evidence and are explicitly qualified as such throughout the text. Overlapping patient populations across meta-analyses were identified by cross-referencing trial lists; where the same trial population contributed to multiple meta-analyses cited in this review, the most methodologically rigorous and most recently updated meta-analysis was used for quantitative estimates, and the overlap is noted in context.

Guideline documents, regulatory agency communications, and expert consensus statements were incorporated solely to provide clinical context, for drug approval status, recommended indications, and standard-of-care framing and were not used as primary evidence sources for efficacy or safety conclusions. All efficacy and safety conclusions in this review are grounded in the RCT and observational evidence hierarchy described above.

Conflicting findings across trials and observational studies were handled according to the following pre-specified approach: (1) where RCT-level meta-analyses and individual trials produced discordant estimates, the meta-analytic estimate was used as the primary citation with individual trial results noted for context; (2) where observational studies conflicted with RCT evidence, RCT findings were treated as primary and observational data cited as hypothesis-generating or for real-world context only; (3) where observational studies produced conflicting estimates among themselves, the direction and magnitude of effect across studies was described narratively, with unmeasured confounding explicitly noted as a likely contributor to heterogeneity; and (4) where a single trial provided the only available evidence for a specific endpoint or population, this limitation is stated in the relevant section and identified as a research gap.

## Mechanisms of cardio-renal protection

GLP-1 receptors (GLP-1R) are expressed on cardiomyocytes, endothelial cells, renal tubular cells, and immune cells [9]. Receptor activation triggers cAMP-dependent Gs protein signaling, activating PKA and MAPK pathways that suppress NF-κB-mediated inflammation and promote cell survival [10]. The mechanisms described below integrate preclinical and early clinical evidence; where data derive solely from animal or in vitro models, this is noted. The precise molecular substrates of GLP-1RA pleiotropic benefit in humans remain incompletely understood.

### Cardiovascular mechanisms

GLP-1RAs enhance endothelial nitric oxide (NO) bioavailability via eNOS activation, an effect demonstrated in human umbilical vein endothelial cell (HUVEC) assays using Western blot quantification of phosphorylated eNOS (p-eNOS Ser1177) and fluorometric NO detection, reducing adhesion molecule expression (ICAM-1, VCAM-1, measured by ELISA and flow cytometry) and monocyte infiltration in both preclinical and early human pharmacodynamic studies [11]. Pro-inflammatory cytokines; TNF-α, IL-6, and IL-1β, are suppressed following GLP-1RA exposure, quantified in human studies via high-sensitivity multiplex immunoassay (Luminex platform) and ELISA from plasma samples of CVOT biomarker sub-studies [12]. The extent to which these biomarker-level effects translate to the observed MACE reductions in CVOTs is likely multifactorial and remains incompletely characterized at the mechanistic level. In the myocardium, preclinical data suggest suppression of TGF-β-mediated fibroblast transdifferentiation and NLRP3 inflammasome activation [13], with potential relevance to HFpEF pathophysiology. GLP-1RAs also augment natriuretic peptide signaling, contributing to blood pressure reduction independently of weight loss in human studies [14].

### Renal mechanisms

In renal tissue, GLP-1RAs promote afferent arteriolar vasodilation via NO, quantified by urinary nitrate/nitrite excretion and renal blood flow measured by para-aminohippurate clearance in early human studies, and natriuresis through natriuretic peptide signaling, alongside suppression of glomerular inflammation via NF-κB and NLRP3 inhibition demonstrated in renal biopsy-derived human mesangial cell cultures and confirmed by UACR reduction as a surrogate endpoint across multiple CVOTs [5, 15]. In addition to afferent arteriolar vasodilation, GLP-1RAs have been proposed to promote efferent arteriolar vasodilation through suppression of angiotensin II, an effect that would reduce intraglomerular pressure and attenuate hyperfiltration, one of the key hemodynamic drivers of progressive glomerular injury in both diabetic and non-diabetic CKD [16]. This efferent mechanism is mechanistically distinct from the efferent vasoconstriction produced by SGLT2 inhibitors (section “Mechanistic basis for combination with SGLT2 inhibitors”, and the two effects may be complementary in reducing intraglomerular hypertension through different vascular segments of the glomerular microcirculation. Suppression of fibroblast transdifferentiation may limit glomerular fibrosis [17], and RAGE signaling suppression has been proposed as a pathway relevant to non-diabetic CKD, though direct human evidence remains limited [18]. The mechanistic pathways described above are the subject of ongoing prospective investigation in human participants. The REMODEL trial (Renal Effects of GLP-1 Receptor Agonist Treatment - a Mechanistic Study) is designed to directly interrogate GLP-1RA renal mechanisms in vivo; a methods article has been published [19], and results are anticipated to substantially advance the characterization of human renal GLP-1R biology.

### Mechanistic basis for combination with SGLT2 inhibitors

GLP-1RAs and SGLT2 inhibitors act through largely complementary mechanisms with some points of divergence. GLP-1RAs target gut-brain-vascular cAMP-dependent signaling and promote afferent arteriolar vasodilation and, potentially, efferent arteriolar vasodilation, reducing intraglomerular pressure through vasodilatory mechanisms. SGLT2 inhibitors reduce intraglomerular hypertension primarily through a distinct hemodynamic reset: tubuloglomerular feedback-mediated afferent arteriolar vasoconstriction, with some evidence suggesting additional efferent arteriolar effects, alongside suppression of NLRP3 inflammasome activation [20, 21]. The net effect of both classes is reduction in intraglomerular hypertension and hyperfiltration, achieved through mechanistically distinct but potentially additive vascular pathways, providing a strong hemodynamic rationale for combination therapy beyond the anti-inflammatory and metabolic complementarity already described. This divergence provides biological rationale for combination therapy.

Preclinical models demonstrate additive albuminuria reduction with dual therapy [22]; prospective human confirmation is needed. Notably, meta-analyses of major SGLT2 inhibitor trials show consistent cardiovascular and renal efficacy regardless of background GLP-1RA use, and similarly, GLP-1RA effects in CVOTs are consistent with and without concomitant SGLT2 inhibitor use [23], providing indirect evidence that the two classes do not substantially attenuate each other’s benefits, a prerequisite for meaningful combination therapy. Beyond hemodynamic complementarity, the two classes diverge in their primary metabolic targets: GLP-1RAs reduce appetite, promote weight loss, suppress postprandial glucagon, and attenuate systemic inflammation through central and peripheral GLP-1R signaling; SGLT2 inhibitors promote glucosuria and osmotic diuresis, reduce cardiac preload and afterload, and shift myocardial substrate utilization toward ketone oxidation. These divergent metabolic mechanisms operate in parallel rather than in competition, providing a multi-pathway rationale for combination that extends beyond the renal hemodynamic complementarity described above. The anti-fibrotic effects of GLP-1RAs (via TGF-β suppression) and the NLRP3 inflammasome suppression shared by both classes may also act additively at the level of glomerular and myocardial remodeling, though direct human evidence for additive anti-fibrotic benefit with combination therapy is currently limited to biomarker substudy data.

### Finerenone: complementary mineralocorticoid receptor antagonism

Finerenone, a non-steroidal selective mineralocorticoid receptor antagonist (MRA), completes the four-pillar mechanistic framework for CKM protection in type 2 diabetes. Unlike GLP-1RAs and SGLT2 inhibitors, which act primarily through metabolic and hemodynamic pathways, finerenone targets the inflammatory and fibrotic consequences of aldosterone-mediated mineralocorticoid receptor overactivation, a distinct mechanism that is additive to both RAAS blockade and the effects of the other drug classes [24].

In the FIDELIO-DKD trial (*n* = 5,734, T2DM + CKD), finerenone reduced the primary composite of kidney failure, sustained ≥ 40% eGFR reduction, or renal death by 18% (HR 0.82; 95% CI 0.73–0.93) and MACE by 14% (HR 0.86; 95% CI 0.75–0.99) [25]. FIGARO-DKD (*n* = 7,437, T2DM + CKD with broader eGFR range) demonstrated a 13% reduction in MACE (HR 0.87; 95% CI 0.76–0.98) and significant reduction in kidney disease progression [26]. A pooled analysis of both trials (FIDELITY, *n* = 13,026) confirmed consistent cardiorenal protection across the full spectrum of CKD severity in T2DM [27]. Most recently, the FINEARTS-HF trial demonstrated that finerenone reduced worsening heart failure events and cardiovascular death in HFmrEF/HFpEF (HR 0.84; 95% CI 0.74–0.95) [28], establishing its role beyond diabetic kidney disease into the HFpEF domain where GLP-1RAs also demonstrate benefit. The CONFIDENCE trial showed that the combination of finerenone and an SGLT2 inhibitor produced greater albuminuria reduction than either agent alone in T2DM + CKD [29], providing proof-of-concept for multi-pillar combination therapy and setting the stage for trials evaluating all three classes together.

## Clinical evidence from major trials

### Cardiovascular outcomes trials in type 2 diabetes

A meta-analysis of nine major CVOTs (ELIXA, LEADER, SUSTAIN-6, EXSCEL, HARMONY, REWIND, PIONEER 6, AMPLITUDE-O, SOUL) involving 69,730 patients with type 2 diabetes demonstrated a 13% reduction in MACE (HR 0.87; 95% CI 0.82–0.93), driven by a 12% reduction in cardiovascular death and a 15% reduction in stroke [30]. A subsequent meta-regression incorporating FLOW and SOUL found that each 1% additional HbA1c reduction was associated with a 27% lower hazard ratio for MACE, while change in body weight was not significantly associated with cardiovascular outcomes in this analysis [31]. This association is hypothesis-generating and should not be interpreted as evidence that glycemic lowering alone drives cardioprotection; meta-regression cannot establish causality, residual confounding is substantial, and the specific contribution of individual mechanisms to observed benefits cannot be disaggregated from trial-level data. A summary of the cardiovascular outcomes in several major trials is shown in Table 1.

**Table 1:** Key trials informing GLP-1RA use across the CKM continuum. summary of population, follow-up, and primary outcomes*

Ref.	Trial	Drug	Population	Follow-up	Primary Endpoint	Key Result	HR (95% CI)
[32]	LEADER	Liraglutide	T2DM +high CV risk	3.8 yr	3-pt MACE	↓ MACE, ↓ CV death	0.87 (0.78–0.97)
[33]	SUSTAIN-6	Semaglutide	T2DM + high CV risk	2.1 yr	3-pt MACE	↓ MACE, ↓ stroke	0.74 (0.58–0.95)
[34]	REWIND	Dulaglutide	Broad T2DM (many without prior CVD)	5.4 yr	3-pt MACE	↓ MACE	0.88 (0.79–0.99)
[35]	HARMONY	Albiglutide	T2DM + established CVD	1.6 yr	3-pt MACE	↓ MACE	0.78 (0.68–0.90)
[36]	AMPLITUDE-O	Efpeglenatide	T2DM + high CV/renal risk	1.8 yr	3-pt MACE	↓ MACE + renal benefit	0.73 (0.58–0.92)
[37]	EXSCEL	Exenatide	T2DM (broad)	3.2 yr	3-pt MACE	Non-inferior	0.91 (0.83-1.00)
[4]	SELECT	Semaglutide	Overweight/obesity, no diabetes + CVD	3.3 yr	3-pt MACE	↓ MACE in non-diabetic population	0.80 (0.72–0.90)
[5]	FLOW	Semaglutide	T2DM + CKD (eGFR 25–75 ml/min/1.73m^2^)	34.6 mo.	Renal composite	↓ kidney failure, ↓ CV events (stopped early for efficacy)	0.76 (0.66–0.88)
[38]	SOUL	Semaglutide	T2DM + ASCVD/CKD	2.6 yr	3-pt MACE	↓ MACE; HF subgroup benefit	0.86 (0.77–0.96)
[39]	STEP-HFpEF (pooled)	Semaglutide	Obesity + HFpEF (LVEF ≥ 45%)	52 wks.	CV death/worsening HF	↓ worsening HF events	0.59 (0.41–0.82)
[40]	SURPASS-CVOT	Tirzepatide	T2DM + established ASCVD	~ 2.4 yr	3-pt MACE	Non-inferior to dulaglutide	0.92 (0.83–1.01)

HR = hazard ratio; CI = Confidence Interval; T2DM = type 2 diabetes mellitus; ASCVD = Atherosclerotic cardiovascular disease; CVD = Cardiovascular disease; CKD = chronic kidney disease; MACE = major adverse cardiovascular event; yr = years; mo. = month; wks. = weeks

*ELIXA (lixisenatide, T2DM + recent ACS, HR 1.02; 95% CI 0.89–1.17, non-inferior) and PIONEER 6 (oral semaglutide, T2DM + high CV risk, HR 0.79; 95% CI 0.57–1.11, non-inferior) are included in the meta-analysis cited [30] but are not shown individually as neither demonstrated significant MACE reduction and their populations overlap substantially with trials listed above

Critical appraisal of these trials requires attention to important design heterogeneity that limits direct cross-trial comparison. Baseline cardiovascular risk varied substantially across CVOTs; HARMONY and AMPLITUDE-O enrolled exclusively high-risk patients with established CVD, while REWIND included approximately 69% of patients without prior Cardiovascular events, a broader and lower-risk population that may partly explain REWIND’s more modest absolute risk reduction despite similar relative effect. Statistical power differed accordingly; trials enrolling lower-risk populations required longer follow-up or larger sample sizes to accrue sufficient events, and several trials (EXSCEL, PIONEER 6) were powered for non-inferiority rather than superiority, limiting conclusions about magnitude of benefit. Background therapy also evolved across the trial era; later trials enrolled higher proportions of patients on statins, RAAS inhibitors, and SGLT2 inhibitors, which may have compressed the absolute risk reduction achievable by adding a GLP-1RA. These design differences mean that hazard ratios should not be compared directly across trials as if they were head-to-head estimates, and that NNT calculations are sensitive to the baseline risk of the population to which they are applied.

### SELECT trial: non-diabetic obesity

The SELECT trial (November 2023) enrolled 17,604 patients with overweight or obesity (BMI ≥ 27 kg/m²) and established ASCVD but without diabetes [4]. Over a median follow-up of 39.8 months, semaglutide 2.4 mg weekly reduced 3-point MACE by 20% (HR 0.80; 95% CI 0.72–0.90; *p* < 0.001) compared with placebo. Cardiovascular benefits were consistent across all baseline weight and waist circumference categories.

A pre-specified exploratory mediation analysis revealed that waist circumference reduction accounted for approximately 33% of the MACE reduction, with the remaining 67% unexplained by measured adiposity and cardiometabolic changes [6]. These findings are hypothesis-generating; they suggest the existence of weight-independent mechanisms but do not identify them. Several methodological limitations of mediation analysis should be noted when interpreting this residual; the analysis was exploratory rather than pre-specified as a primary endpoint, mediation models assume no unmeasured confounding between mediators and the outcome, estimates are sensitive to measurement error in the mediators, and dynamic biological processes such as cumulative endothelial repair or vascular remodeling may not be captured by static biomarker measurements. These limitations mean the 67% unexplained fraction is best understood as a lower bound on the potential contribution of pleiotropic mechanisms, not a precise estimate of their magnitude. Clinicians should not use the unexplained residual fraction from this exploratory analysis to justify GLP-1RA use in populations or for indications not supported by primary RCT evidence.

### FLOW trial: dedicated renal outcomes

The FLOW trial (2024) was the first large-scale, prospective, dedicated kidney outcomes trial of a GLP-1RA [5]. It enrolled 3,534 patients with type 2 diabetes and CKD (eGFR 25–75 mL/min/1.73 m^2^) on background RAAS inhibition, randomized to semaglutide 1.0 mg weekly or placebo. The primary composite endpoint; kidney failure, ≥ 50% eGFR reduction, or kidney or cardiovascular death, was reduced by 24% (HR 0.76; 95% CI 0.66–0.88; NNT 17 over 34.6 months). The trial was stopped early for efficacy. Secondary outcomes included 18% MACE reduction (HR 0.82; 95% CI 0.68–0.98), 20% all-cause mortality reduction (HR 0.80; 95% CI 0.67–0.95), and 1.16 mL/min/1.73 m^2^/year slower eGFR decline versus placebo [5, 15]. These findings position GLP-1RAs as a fourth pillar of diabetic kidney disease management alongside RAAS inhibitors, SGLT2 inhibitors, and finerenone, consistent with the four-pillar framework proposed for comprehensive cardiorenal protection in type 2 diabetes [41].

Important evidence gaps persist, however, and intellectual honesty requires naming them alongside the trial successes. Renal outcomes data from FLOW apply exclusively to patients with type 2 diabetes and CKD; whether comparable kidney protection exists in non-diabetic CKD remains untested in a dedicated outcomes trial. Heart failure data are strongest in HFpEF with obesity; the role of GLP-1RAs in HFrEF, where SGLT2 inhibitors are clearly superior, remains limited. Cardiovascular outcomes in non-White populations are directionally consistent but statistically underpowered due to enrollment disparities in the trials themselves [42]. The mechanistic basis of the majority of cardiovascular benefit, as suggested by SELECT mediation analysis, remains uncharacterized at the molecular level. And the optimal combination strategy and sequencing with SGLT2 inhibitors has not been evaluated in a prospective randomized trial. These gaps define the research agenda, not the limits of current clinical application.

Knowledge Gap; Kidney outcomes in non-diabetic populations remain investigational. FLOW enrolled exclusively patients with type 2 diabetes and CKD; SELECT demonstrated UACR reduction as a secondary endpoint but was not powered for kidney failure events. The renal benefits of GLP-1RAs in non-diabetic CKD are biologically plausible but clinically unestablished in dedicated outcomes trials. Preliminary evidence from the SMART trial, which enrolled patients with CKD without diabetes, demonstrated a significant reduction in UACR with semaglutide, providing early proof-of-concept for GLP-1RA renoprotection beyond the diabetic population [43]. However, SMART was not powered for hard kidney endpoints, and results should not be extrapolated to clinical practice pending a dedicated outcomes trial. Clinicians should also not extrapolate FLOW findings to non-diabetic patients, and dedicated trials in this population are an urgent research priority.

### Heart failure: evidence in HFpEF

Earlier concerns about GLP-1RA safety in heart failure have been substantially revised. The STEP-HFpEF and STEP-HFpEF DM trials enrolled patients with obesity-related Heart failure with preserved ejection fraction (HFpEF) (LVEF ≥ 45%); their pooled analysis with SELECT and FLOW (3,743 HFpEF participants) demonstrated that semaglutide reduced cardiovascular death or worsening heart failure events by 31% (HR 0.69; 95% CI 0.53–0.89; *p* = 0.0045) and worsening heart failure events alone by 41% (HR 0.59; 95% CI 0.41–0.82) [44]. No significant reduction in cardiovascular death alone was observed, indicating the primary benefit is symptomatic and hospitalization-related. The SOUL trial HF substudy corroborates this finding using the oral formulation of semaglutide, with a 22% reduction in the 3-point HF composite (HR 0.78; 95% CI 0.63–0.96) and 41% reduction in HFpEF specifically [45]. The consistency between injectable and oral semaglutide across STEP-HFpEF and SOUL supports a class effect rather than a formulation-specific benefit, and suggests oral semaglutide may be a clinically equivalent option for patients who prefer or require non-injectable therapy, though this comparison is indirect and no head-to-head trial of oral versus injectable semaglutide for HF outcomes has been conducted. Oral semaglutide does not yet carry an indication for non-diabetic populations.

A network meta-analysis of 39 HFpEF trials (48,235 patients) ranked GLP-1RAs highest (P-score 0.871) for cardiovascular death and HF hospitalization composite (HR 0.73; 95% CI 0.61–0.88), with unique superiority in functional outcomes; 6-minute walk distance + 17.6 m and KCCQ-CSS + 7.38 points [46]. In HFrEF, SGLT2 inhibitors retain superior efficacy (HR 0.86; 95% CI 0.77–0.97). This divergence is clinically important; GLP-1RAs and SGLT2 inhibitors are not interchangeable in heart failure, they occupy distinct niches defined by ejection fraction and the relative contribution of obesity and metabolic inflammation to the HF phenotype. Both GLP-1RAs and SGLT2 inhibitors demonstrate benefit in HFpEF, SGLT2 inhibitors reduced the composite of cardiovascular death or worsening HF in EMPEROR-Preserved (HR 0.79; 95% CI 0.69–0.90) [47]; but GLP-1RAs appear to have particular advantages in obesity-predominant HFpEF, where weight-dependent and metabolic mechanisms contribute substantially to the HF phenotype, and where functional outcome benefits (6-minute walk distance, KCCQ scores) are more consistently demonstrated with GLP-1RA therapy.

### Number needed to treat, real-world effectiveness, and clinical benchmarking

A meta-analysis of 109,846 patients across 25 studies estimated that GLP-1RAs prevent one MACE event per 67 patients treated (NNT 67), one cardiovascular death per 103 patients, and one stroke per 143 patients over approximately 3.5 years [48]. The FLOW kidney NNT of 17 over 34.6 months is substantially more favorable and comparable to established preventive therapies such as Angiotensin converting enzyme inhibitors (ACEi) and statins. Real-world UK data corroborate trial findings, showing a 33% lower adjusted MACE risk for GLP-1RAs versus DPP-4 inhibitors (95% CI 24–40%) and approximately £208 in cardiovascular cost savings per patient-year [49]. Taken together, these NNTs are not merely statistically significant, they are clinically competitive with the most widely prescribed preventive therapies in cardiovascular medicine, and they accrue in patient populations who are already on those therapies. The case for GLP-1RAs as a standard rather than supplementary component of high-risk cardiometabolic management rests on this foundation.

### Safety profile

The safety evidence for GLP-1RAs varies by outcome type; the following distinguishes RCT-confirmed findings from observational signals and anecdotal reports.

Gastrointestinal adverse events (nausea, vomiting, diarrhea) are the most consistently RCT-documented side effects, occurring in the majority of patients during dose escalation and generally resolving with continued therapy [48]. Real-world discontinuation due to gastrointestinal intolerance is estimated at 8–12%, higher than trial rates of approximately 5%, likely reflecting faster titration and less structured monitoring in routine care [50]. This is an observational estimate subject to confounding.

Hypoglycemia risk is RCT-confirmed to be low with GLP-1RA monotherapy. In major CVOT meta-analyses, no significant increase in severe hypoglycemia was observed versus placebo [48]. When combined with insulin or sulfonylureas, dose reductions of 25–50% are recommended. A real-world observational study estimated 70% lower hypoglycemia risk versus sulfonylureas in CKD populations [51]; this finding is directionally consistent with trial data but subject to confounding inherent to observational designs. In a systematic review and meta-analysis of RCTs evaluating GLP-1RAs with and without SGLT2 inhibitors, serious adverse event rates including ketoacidosis were low and comparable across treatment groups, though the combination warrants clinical monitoring especially during periods of reduced caloric intake [23].

Pancreatitis, pancreatic cancer, and retinopathy; Large RCT-level meta-analyses and long-term CVOT data have not demonstrated increased risks of acute pancreatitis, chronic pancreatitis, pancreatic cancer, or retinopathy worsening [48]. These were pre-specified safety outcomes in multiple trials and the evidence against elevated risk is robust.

Nonarteritic anterior ischemic optic neuropathy (NAION) has emerged as an observational safety signal warranting attention. A pharmacovigilance analysis of real-world data identified a potential association between GLP-1RA use and NAION, with an estimated reporting odds ratio suggesting a signal above background rates [52]. This is an observational signal from spontaneous reporting databases and does not establish causation; confounding by indication (patients with cardiometabolic risk factors are independently at higher NAION risk) cannot be excluded. No CVOT has identified NAION as a pre-specified outcome. Clinicians should be aware of this emerging signal and patients with pre-existing optic disc abnormalities or prior NAION episodes may warrant ophthalmologic discussion before initiating therapy, though the absolute risk remains unclear.

Hair shedding is an anecdotal and observational signal. A cross-sectional survey reported a prevalence of 55–70% among GLP-1RA users [53], markedly higher than the 3–7% observed in trials. The effective mechanism is likely due to telogen effluvium from rapid weight loss [54]. This discrepancy is likely attributable to survey methodology, recall bias, voluntary reporting, and temporal association with concurrent rapid weight loss (≥ 15%) rather than a direct drug effect. Prospective data on prevalence, causality, temporal course, and resolution are absent. Patients should be counseled that hair shedding has been reported and may be transient, while acknowledging that this signal is not RCT-confirmed.

## Combination therapy: GLP-1RAs and SGLT2 inhibitors

Given their mechanistically complementary profiles (section “Mechanistic basis for combination with SGLT2 inhibitors”), the combination of GLP-1RAs and SGLT2 inhibitors has been evaluated across three evidence tiers that must be weighted with particular caution. At the RCT level, no dedicated trial has randomized patients to combination versus monotherapy as a primary question, nor has any prospective trial compared sequential versus simultaneous initiation strategies. This is a fundamental evidentiary gap: all clinical conclusions about the benefit of combination therapy in this section derive from observational data or post-hoc analyses of trials not designed to test this question. The real-world observational evidence is consistently directionally favorable but subject to substantial and likely irreducible confounding; patients receiving combination therapy are systematically different from those on monotherapy, being more engaged with care, of higher socioeconomic status, and more likely to be managed by specialist-involved multidisciplinary teams. These differences are difficult to fully adjust for even in propensity-matched analyses. Mechanistic support is present, the complementary pathways described in section “Mechanistic basis for combination with SGLT2 inhibitors” provide biological plausibility for additive benefit, but preclinical synergy does not reliably predict clinical outcomes. The following synthesis should therefore be interpreted as generating hypotheses for prospective RCT evaluation, not as establishing combination therapy as an evidence-based standard of care equivalent to monotherapy indications with dedicated trial support.

### Cardiovascular and heart failure outcomes

In a propensity-matched cohort of 58,525 patients with heart failure and type 2 diabetes, dual therapy was associated with lower all-cause mortality (HR 0.65; 95% CI 0.60–0.70) and acute myocardial infarction (HR 0.80; 95% CI 0.71–0.89) compared with SGLT2 inhibitor monotherapy [55]. A meta-analysis of 10 RCTs found combination therapy reduced heart failure hospitalization by 63% versus monotherapy [56], while a systematic review and meta-analysis of cohort studies (1,164,774 participants) found lower MACE risk (RR 0.56) and all-cause mortality (RR 0.50) with combination versus monotherapy [55]. These findings are hypothesis-generating. The magnitude of benefit reported in observational combination therapy analyses, including the 63% heart failure hospitalization reduction in the RCT meta-analysis, should be interpreted in light of the small number of contributing trials, heterogeneity in background therapy, and the inability of observational designs to exclude channeling bias toward combination therapy in clinically healthier or more adherent patients.

### Renal outcomes

In a target trial emulation including 504,151 individuals with type 2 diabetes, combination therapy reduced kidney failure risk by 28% compared with GLP-1RA monotherapy, with greater benefit (46% risk reduction) in those with baseline CKD or heart failure [57]. A post-hoc analysis of the RECAP study found that adding a GLP-1RA after SGLT2 inhibitor initiation improved annual eGFR decline from − 3.5 to -0.4 mL/min/1.73 m^2^/year (*p* < 0.001) [58], for context, substantially exceeding the 1.16 mL/min/1.73 m^2^/year eGFR preservation demonstrated by semaglutide monotherapy in FLOW (section “FLOW trial: dedicated renal outcomes”). This comparison is illustrative and not formally valid across different study populations, but it underscores the potential magnitude of renal benefit with optimized dual therapy, pending prospective validation. The eGFR improvement estimate from RECAP is from a post-hoc analysis of a non-randomized cohort and should not be compared directly with FLOW monotherapy data; the populations, follow-up durations, and study designs differ in ways that preclude meaningful cross-study quantitative comparison.

### Comparative effectiveness: GLP-1RAs vs. SGLT2 inhibitors

No head-to-head RCT comparing the two classes is available. A network meta-analysis of 23 CVOTs found no significant MACE difference (GLP-1RA HR 0.87 vs. SGLT2 inhibitor HR 0.88 vs. placebo) [58], but GLP-1RAs uniquely reduced stroke (HR 0.85), while SGLT2 inhibitors showed superior heart failure hospitalization reduction (HR 0.74) and renal outcome protection. Class selection should therefore be guided by the dominant comorbidity; stroke history or obesity-related HFpEF favors GLP-1RA, HFrEF or CKD without high cardiovascular risk favors SGLT2 inhibitor, high-risk patients with multiple comorbidities are most likely to benefit from combination. The emerging four-pillar model of CKM protection; RAAS inhibitors, SGLT2 inhibitors, GLP-1RAs, and finerenone, recognizes that each class targets distinct but complementary pathways, and that the greatest organ protection is likely achieved through rational combination rather than class substitution. The CONFIDENCE trial provides early evidence that finerenone and SGLT2 inhibitor combination produces additive albuminuria reduction beyond either agent alone; prospective trials evaluating GLP-1RA added to this backbone are a high-priority research agenda item. Table 2 summarizes these distinctions.

**Table 2:** Summary of GLP-1RA efficacy across key clinical indications

Indication	Evidence Type	MACE/CV Outcome	Renal Outcome	HF Outcome	NNT	Agent with Evidence
T2DM + ASCVD	RCT meta-analysis	HR 0.87 (95% CI 0.82–0.93) − 13% reduction	Not primary	Not primary	67 (MACE)	Semaglutide 1 mg; Dulaglutide 1.5 mg
T2DM + CKD	RCT (FLOW)	HR 0.82 (95% CI 0.68–0.98) − 18% reduction	HR 0.76 (95% CI 0.66–0.88) − 24% reduction	Not primary	17 (Kidney)	Semaglutide 1 mg
T2DM + HFpEF	RCT pooled	HR 0.69 (95% CI 0.53–0.89) − 31% reduction	UACR reduction (no dedicated renal composite endpoint in STEP-HFpEF trials)	HR 0.59 (95% CI 0.41–0.82) − 41% reduction in worsening HF events	12–15 (HF events)	Semaglutide 2.4 mg
Non-DM Obesity + ASCVD	RCT (SELECT)	HR 0.80 (95% CI 0.72–0.90) − 20% reduction	UACR reduction (secondary endpoint; not powered for kidney failure events)	Not primary	50 (MACE)	Semaglutide 2.4 mg

HR = hazard ratio; NNT = number needed to treat over trial duration; T2DM = type 2 diabetes mellitus; ASCVD = Atherosclerotic cardiovascular disease; CKD = chronic kidney disease; MACE = major adverse cardiovascular events. Evidence is most robust for T2DM populations. Non-diabetic renal and HFrEF data are limited

### Sequencing considerations

Two sequencing strategies are supported by observational data. Sequential initiation; SGLT2 inhibitor first, with GLP-1RA addition after approximately 3 months, is supported by the RECAP post-hoc analysis for CKD stages 3–4, though prospective trial confirmation is needed. Simultaneous initiation is suggested by real-world data in patients with ASCVD, obesity, and advanced CKD, though dual-therapy discontinuation rates of approximately 49% versus 22% for monotherapy, driven largely by gastrointestinal effects, represent a meaningful practical concern [59]. In routine practice, cost and insurance coverage frequently govern sequencing decisions more than mechanistic rationale [59].

## Comparative effectiveness: GLP-1RAs vs. tirzepatide

Tirzepatide is a dual Glucose-Dependent Insulinotropic Polypeptide (GIP) and GLP-1 receptor agonist achieving superior glycemic and weight outcomes versus GLP-1RA monotherapy; HbA1c reductions of up to 2.58% versus 1.24% for semaglutide and greater weight loss across the SURPASS program [60], through complementary GIP receptor activation enhancing insulin secretion, insulin sensitivity, and triglyceride reduction [61, 62]. Two major developments published in late 2025 have substantially clarified the cardiovascular evidence base for tirzepatide.

SURPASS-CVOT (December 2025) was a pre-specified, double-blind, active-comparator RCT enrolling 13,299 patients with type 2 diabetes and established ASCVD, randomized to tirzepatide (up to 15 mg weekly) versus dulaglutide (1.5 mg weekly) [48]. The primary composite endpoint; cardiovascular death, myocardial infarction, or stroke, occurred in 12.2% of tirzepatide patients versus 13.1% of dulaglutide patients (HR 0.92; 95% CI 0.83–1.01), confirming non-inferiority (*p* = 0.003) but not superiority (*p* = 0.09). Gastrointestinal adverse events were more frequent with tirzepatide. These results establish tirzepatide as cardiovascularly non-inferior to dulaglutide in this population, without demonstrating superiority on hard cardiovascular endpoints.

Real-world HFpEF comparison (October 2025); five propensity-weighted cohort studies using US claims data (up to 28,100 patients in the tirzepatide vs. semaglutide comparison) found that tirzepatide had no meaningfully lower risk of heart failure hospitalization or all-cause mortality compared with semaglutide in cardiometabolic HFpEF (HR 0.86; 95% CI 0.70–1.06) [63]. Both agents showed substantial benefit versus sitagliptin as a placebo proxy (HR 0.58 for semaglutide; HR 0.42 for tirzepatide). A separate real-world analysis in non-diabetic patients with obesity and ASCVD found semaglutide associated with lower MACE risk versus tirzepatide [64], reflecting that semaglutide currently holds the only regulatory indication for cardiovascular risk reduction in non-diabetic obesity.

For renal outcomes, tirzepatide slowed eGFR decline more than insulin glargine in SURPASS-4 (-1.4 vs. -3.6 mL/min/1.73 m^2^/year) and reduced UACR versus an increase with insulin [37]. No dedicated prospective kidney outcomes trial for tirzepatide has been completed, in contrast to FLOW for semaglutide.

### Clinical positioning

Updated with SURPASS-CVOT (December 2025), tirzepatide is cardiovascularly non-inferior to dulaglutide in T2DM with ASCVD and achieves superior glycemic and weight reduction versus GLP-1RA monotherapy across the SURPASS program.

### Appropriate use

Tirzepatide is a reasonable alternative to established GLP-1RAs when superior glycemic or weight reduction is the primary therapeutic goal, or in patients who have not achieved adequate metabolic control on GLP-1RA monotherapy.

### Cautions and limitations

Tirzepatide should not be substituted for semaglutide or dulaglutide solely on the basis of assumed cardio-renal superiority. Cardiovascular superiority over established GLP-1RAs has not been demonstrated, no dedicated kidney outcomes trial for tirzepatide has been completed, and semaglutide retains the only regulatory indication for MACE reduction in non-diabetic obesity. Clinicians should apply the same evidence hierarchy to tirzepatide as to any agent, superiority claims require superiority trial evidence.

## Sex, racial, and ethnic disparities

Women constitute approximately one-third of CVOT participants, a recognized underrepresentation, yet GLP-1RA cardiovascular benefits appear broadly consistent between sexes. In T2DM trials, MACE reduction was similar in men (HR 0.89; 95% CI 0.82–0.96) and women (HR 0.83; 95% CI 0.77–0.91), with a slightly greater but non-significant relative reduction in women [65, 66]. Across racial groups, benefits were consistent in White and Asian populations, with Asian patients showing somewhat greater relative MACE reduction (HR 0.73; 95% CI 0.63–0.85 vs. HR 0.86; 95% CI 0.81–0.91) [67]. In Black/African American patients, the direction of benefit was consistent but did not reach statistical significance (HR 0.88; 95% CI 0.67–1.15), most likely reflecting underrepresentation rather than differential efficacy [66]. SELECT enrolled approximately 80% White participants, substantially limiting generalizability to diverse populations [42]. Compounding this enrollment disparity, a 2026 genome-wide association study of 27,885 GLP-1RA users identified that the GLP1R efficacy allele (rs10305420) is least common in African ancestry populations (7% minor allele frequency versus 40% in Europeans), raising the possibility that population-level differences in pharmacogenomic response may further widen observed outcome disparities, though this requires prospective clinical validation [68].

These equity gaps extend directly into prescribing practice; Black patients receive GLP-1RAs 20–30% less often than White patients, Hispanic patients 9–34% less often, with rural and low-income populations facing compounded barriers [69]. Provider-level bias, historical medical mistrust, linguistic barriers, and geographic access limitations compound cost and insurance barriers as drivers [70]. Closing this prescribing gap is not only an equity imperative but a public health priority given the disproportionate cardiometabolic disease burden in affected communities.

## Implementation: barriers, patient selection, and clinical practice

### A comorbidity-guided clinical decision framework

The CKM Disease-Modification Framework proposed in this review rests on a single organizing principle; clinical decisions about GLP-1RA therapy should be driven by the patient’s dominant organ risk, not their HbA1c or diabetes status. Translating this into practice requires a decision lens organized by comorbidity burden rather than drug class, with sequencing guided by where organ protection is most urgently needed.

The framework proposed here is consistent with, and informed by, current major clinical practice guidelines. The 2024 ADA Standards of Care recommend GLP-1RAs with proven cardiovascular benefit in patients with T2DM and established ASCVD or high cardiovascular risk, independently of HbA1c [71]. The 2022 KDIGO Diabetes Management in CKD guideline recommends GLP-1RAs as second-line agents after SGLT2 inhibitors in patients with T2DM and CKD, with the combination preferred in high-risk patients [72]. The 2023 ESC Guidelines on Diabetes and Cardiovascular Disease recommend GLP-1RAs in patients with T2DM and established CVD or high/very high CV risk, with a Class I, Level A recommendation for agents with proven CV benefit [73]. Where the CKM Disease-Modification Framework extends beyond current guidelines is in its explicit comorbidity-guided sequencing logic and its incorporation of the four-pillar model including finerenone, areas where guideline updates are anticipated as the evidence base continues to mature.

The following framework reflects the current RCT evidence hierarchy:

- Dominant comorbidity: ASCVD + type 2 diabetes → Either GLP-1RA or SGLT2 inhibitor is appropriate as the preferred add-on to metformin, with comparable MACE reduction across network meta-analyses (GLP-1RA HR 0.87 vs. SGLT2 inhibitor HR 0.88 vs. placebo [58]); selection should be guided by dominant organ risk. GLP-1RA is preferred when stroke history, obesity, or obesity-related HFpEF predominates (RCT evidence: LEADER, REWIND, HARMONY, AMPLITUDE-O; NNT 67 for MACE). SGLT2 inhibitor is preferred when heart failure hospitalization prevention or hemodynamic renal protection is the priority. Combination is appropriate in high-risk patients with multiple comorbidities.
- Dominant comorbidity: Diabetic CKD (eGFR 25–75) → SGLT2 inhibitor first-line for hemodynamic renal protection, then add GLP-1RA after 3 months if albuminuria persists (FLOW evidence base; NNT 17 for kidney composite over 34.6 months). This sequencing is observational, not RCT-proven.
- Dominant comorbidity: Diabetic CKD + persistent albuminuria on RAAS inhibitor + SGLT2 inhibitor → Add finerenone for additional anti-inflammatory and anti-fibrotic renal and cardiovascular protection (FIDELIO-DKD, FIGARO-DKD evidence base). GLP-1RA may then be layered for additional MACE and eGFR benefit per the four-pillar framework, though prospective RCT evidence for all three classes combined is currently limited to the CONFIDENCE trial’s albuminuria endpoint.
- Dominant comorbidity: HFpEF + obesity + type 2 diabetes → GLP-1RA preferred for functional improvement and HF event reduction (STEP-HFpEF, STEP-HFpEF DM, pooled HFpEF analysis; HR 0.59 for worsening HF events). SGLT2 inhibitor adds complementary benefit. Finerenone may be considered for additional HF event reduction where HFmrEF/HFpEF and CKD coexist (FINEARTS-HF evidence base [28]).
- Dominant comorbidity: Obesity + ASCVD without diabetes → Semaglutide 2.4 mg (SELECT evidence; NNT 50 for MACE over 39.8 months). This is currently the only GLP-1RA with regulatory approval for MACE reduction in non-diabetic patients.
- High-risk patients with multiple comorbidities (ASCVD + CKD + HFpEF + obesity) → Combination GLP-1RA + SGLT2 inhibitor represents the evidence-supported standard, with class selection guided by the dominant organ at highest near-term risk.

This framework is intentionally decision-directed rather than comprehensively exhaustive. Evidence is most robust in type 2 diabetes populations. Benefit in non-diabetic CKD, HFrEF, and Metabolic dysfunction-associated steatohepatitis (MASH) remains plausible but not established in dedicated outcome trials, and these indications should not be extrapolated from existing trial populations.

A practical approach to integrating GLP-1 receptor agonists across the CKM continuum may be operationalized in four steps- (1) Early identification, screen all patients with type 2 diabetes, obesity, CKD, or HFpEF for GLP-1RA eligibility at each clinical encounter, using the comorbidity framework above rather than HbA1c thresholds alone; (2) Risk stratification, prioritize patients with the highest predicted absolute benefit using dominant comorbidity, NNT benchmarks, and baseline renal and cardiovascular risk; (3) Therapy initiation and titration, start at the lowest available dose, escalate slowly over 4–6 weeks, adjust concurrent insulin or sulfonylurea doses downward by 25–50%, and sequence SGLT2 inhibitor before GLP-1RA in CKD stages 3–4 where both are indicated; (4) Longitudinal monitoring, assess response at 12–16 weeks (HbA1c, weight, eGFR, UACR). Importantly, the absence of glycemic or weight response does not necessarily indicate treatment failure, cardiovascular and renal benefits in CVOTs are observed across a broad range of metabolic responses and appear partially independent of measured risk factor changes. Dose escalation or formulation change may be considered for inadequate metabolic response, but discontinuation solely on glycemic grounds risks forfeiting established cardio-renal protection. Long-term maintenance planning is essential given the high rate of weight regain after discontinuation. This stepwise approach translates the trial evidence into a repeatable clinical workflow applicable across cardiology, nephrology, endocrinology, and primary care settings.

Knowledge Gap- Non-Diabetic CKD; GLP-1RA renal benefits established in FLOW apply exclusively to patients with type 2 diabetes and CKD. SELECT demonstrated UACR reduction as a secondary endpoint in non-diabetic patients with obesity, but the trial was not designed or powered to detect kidney failure events. GLP-1RA use for renal protection in non-diabetic CKD is currently investigational. Clinicians should not extrapolate FLOW’s NNT of 17 to non-diabetic CKD populations.

### Initiation, titration, and monitoring

Initiation should begin at the lowest available dose with slow escalation over 4–6 weeks to minimize gastrointestinal side effects. Concurrent insulin or sulfonylurea doses should be reduced by 25–50% to mitigate hypoglycemia risk. In CKD stages 3–4, available evidence favors initiating SGLT2 inhibitor therapy first (where indicated) and adding GLP-1RA after 3 months if albuminuria persists or eGFR decline continues, though this recommendation is based on observational data [74].

Monitoring should include fasting glucose, eGFR, and UACR at baseline and at 3-month intervals. Routine laboratory monitoring during SGLT2 inhibitor therapy is not supported by robust evidence; however, in patients concurrently receiving SGLT2 inhibitors alongside ACEi or ARB, periodic electrolyte and renal function checks are a reasonable precaution given the additive natriuretic and hemodynamic effects of these combinations, particularly in CKD stages 3–4 where the risk of hyperkalemia and acute eGFR dipping is higher [75]. Treatment failure should be considered when- HbA1c reduction is < 0.5% after 12–16 weeks; weight loss is < 5% by 24 weeks; annual eGFR decline exceeds 4 mL/min/1.73 m^2^; or side effects are intolerable [76, 77]. Weight regain of 50–65% occurs by 12 months following discontinuation, underscoring the need for long-term maintenance strategies [78].

### Real-world barriers to adoption

Despite strong trial evidence, real-world GLP-1RA uptake remains approximately 8–14% of eligible patients [49]. The primary barriers are fourfold. Cost is prohibitive for many patients, with annual out-of-pocket expenses reaching $1,000-$1,350 in the United States and £99-£450 in the United Kingdom [79, 80]. Insurance and prior authorization structures compound this, as reimbursement frequently requires documented diabetes or prior therapy failure, criteria that exclude non-diabetic ASCVD patients who are eligible per SELECT. Physician awareness remains inadequate, with only 48% of surveyed physicians recognizing cardiovascular benefits in diabetic patients and 39% in non-diabetic obesity [7]. Finally, gastrointestinal tolerability drives discontinuation in approximately 8–12% of real-world users, compared with approximately 5% in trials [50], a gap that likely reflects faster dose titration and less structured monitoring outside clinical trial settings.

### Proposed strategies to improve access and equity

The following strategies have been proposed as responses to documented GLP-1RA prescribing disparities. Direct evidence from implementation trials demonstrating that these interventions close racial, ethnic, or socioeconomic gaps in GLP-1RA access is currently limited; these approaches represent a reasonable framework for action warranting rigorous prospective evaluation rather than a menu of evidence-based recommendations. Policy reform, including insurance coverage expansion to encompass non-diabetic ASCVD indications as established by SELECT, pricing regulation, and mandated equity monitoring in prescribing data, is a logical starting point, though the comparative effectiveness of specific instruments such as price regulation versus benefit expansion versus prior authorization reform has not been formally evaluated in this context. Provider education, particularly structured continuing medical education on cardio-renal benefits independent of glycemic targets, is especially needed in primary care settings, where the majority of eligible patients are managed but GLP-1RA uptake remains lowest [81].

Multidisciplinary care teams and digital health platforms may improve adherence and access in resource-limited settings, though evidence specific to closing GLP-1RA prescribing disparities is largely inferential rather than trial-proven. Finally, culturally adapted implementation studies in underserved, rural, and minority-majority communities are urgently needed to generate the evidence base that currently does not exist for targeted GLP-1RA equity interventions [70, 82].

## Future directions and research priorities

### Mechanistic research

The most fundamental gap is characterization of the pleiotropic mechanisms that exploratory mediation analyses suggest may account for a substantial fraction of GLP-1RA cardiovascular benefit. Priority research includes biomarker-guided mechanistic substrate trials dissecting endothelial, anti-inflammatory, natriuretic, and anti-fibrotic pathways independently of weight change; cardiac MRI and single-cell transcriptomic studies [83]; and multi-omics integration to characterize GLP-1R signaling networks. Without such trials, the mechanistic basis of benefit will remain, as it is now, incompletely understood.

### Precision medicine and pharmacogenomics

GLP-1RAs exhibit marked inter-individual variability in glycemic, weight, and cardiovascular response [84]. The largest pharmacogenomic study to date, a genome-wide association study of 27,885 GLP-1RA users, identified a missense variant in GLP1R (rs10305420, p.Pro7Leu) significantly associated with greater weight loss efficacy (*P* = 2.9 × 10^− 10^; approximately − 0.76 kg per effect allele), with variants in both GLP1R and GIPR independently associated with nausea and vomiting. Notably, the GIPR vomiting association was restricted to tirzepatide users, consistent with the hypothesis that GIP receptor activation attenuates GLP-1-mediated nausea, and the efficacy allele was substantially less common in African ancestry populations (7% vs. 40% in Europeans), compounding existing prescribing disparities. Earlier genome-wide studies have also identified variants in TCF7L2 and MC4R associated with differential weight-loss response [68, 85]. Diabetes phenotyping algorithms (SIDD, SIRD, MOD, MARD) suggest differential response profiles [86]. AI-driven stratification models integrating electronic health records and multi-omics data show promise for identifying optimal responders [87]. All such approaches remain experimental and require prospective clinical validation before adoption.

### Combination and sequencing trials

Prospective RCTs comparing SGLT2 inhibitor-first versus GLP-1RA-first versus simultaneous initiation across CKD stages are a high priority. The RECAP post-hoc analysis [74] provides a testable hypothesis; dedicated prospective evidence is needed. Trials should pre-specify renal, cardiovascular, and patient-reported outcome endpoints and enroll racially and ethnically diverse populations.

### Long-term outcome data for newer agents

SURPASS-CVOT established tirzepatide’s non-inferiority to dulaglutide for MACE in T2DM with ASCVD, but superiority was not demonstrated [40]. A dedicated kidney outcomes trial for tirzepatide remains a priority, as does a cardiovascular outcomes trial in non-diabetic obesity. Retatrutide (GLP-1/GIP/glucagon triple agonist, achieving ~ 22–25% weight loss in Phase 2) [88] requires dedicated cardiovascular and renal outcomes trials before clinical positioning can be established. Oral GLP-1RA formulations (orforglipron, oral semaglutide; 11-13.6% weight loss) [89] may expand access beyond injectable-tolerant populations, but cardiovascular and renal outcome data remain pending.

### Special populations, equity, and discontinuation

Dedicated outcome trials are needed in non-diabetic CKD, HFrEF, and MASH. Trials in low- and middle-income countries, where cardiometabolic disease burden is highest and access most limited, are urgently needed [90]. Trial enrollment targets should explicitly address documented underrepresentation of racial/ethnic minorities in existing CVOTs [42]. Strategies for lean mass preservation during weight loss and for mitigating weight regain after GLP-1RA discontinuation represent additional unmet research priorities [78].

## Conclusion

The evidence reviewed in this paper collectively supports a conclusion that is both simple and consequential; GLP-1 receptor agonists are no longer best understood as glucose-lowering drugs. The weight of RCT evidence suggests they function as CKM protective agents whose benefits in high-risk populations are large, consistent, and substantially independent of glycemic control. SELECT established that these benefits extend to non-diabetic patients with obesity and established ASCVD. FLOW established dedicated renal outcomes benefit in diabetic CKD that positions GLP-1RAs alongside RAAS inhibitors, SGLT2 inhibitors, and finerenone as a fourth pillar of management. Taken together, this body of evidence supports repositioning GLP-1RAs as a foundational component of cardiometabolic care rather than an adjunct to it, though the precise mechanisms mediating this protection, and its full applicability across non-diabetic populations, require further prospective investigation.

What makes this repositioning intellectually significant is the question it raises about mechanisms: in SELECT, a substantial fraction of cardiovascular benefit cannot be accounted for by changes in measured risk factors, as suggested by exploratory mediation analyses. This is not merely a statistical curiosity, it suggests that current biomarker frameworks may not fully capture the pathways through which GLP-1RAs confer protection. These findings are hypothesis-generating and do not establish specific causal mechanisms; they do, however, justify ongoing mechanistic investigation and caution against reducing GLP-1RA benefit to any single biological pathway. We propose a CKM disease-modification paradigm in which GLP-1 receptor agonists exert coordinated, multi-organ protective effects across the CKM continuum, effects that are independent of and additive to, traditional risk factor management. Until prospective mechanistic trials characterize the specific pathways mediating this protection, clinicians should resist reducing GLP-1RA benefit to any single mechanism, and equally resist withholding therapy in patients whose measured risk factors appear adequately controlled.

The combination of GLP-1RAs with SGLT2 inhibitors represents the next frontier of evidence development. Observational data are consistently encouraging, SGLT2 inhibitor-first sequencing in advanced CKD has biological and empirical support, and the mechanistic rationale for additive benefit is strong. But RCT-level sequencing evidence does not exist yet, and this gap should be treated as the field’s most urgent randomized trial priority. SURPASS-CVOT’s confirmation that tirzepatide is non-inferior to dulaglutide is a meaningful step, but it does not establish tirzepatide superiority over established GLP-1RAs, a distinction clinicians must hold carefully as commercial messaging intensifies.

The implementation gap is where population-level impact is currently being lost. Real-world uptake of 8–14% of eligible patients, in the face of NNTs comparable to statins and ACE inhibitors, represents a systemic failure, not a scientific one. The barriers are known; cost, prior authorization structures designed for a glycemic-control era, and physician awareness calibrated to the same. The CKM Disease-Modification Framework proposed here is designed to shift that calculus; by orienting clinical decision-making around dominant organ risk rather than glycemic status, it creates a rationale for earlier, broader GLP-1RA deployment that is grounded in the evidence and actionable in practice. Recognizing GLP-1 receptor agonists as CKM disease-modifying therapies may fundamentally reshape cardiometabolic care, shifting treatment paradigms from glucose-centric control to integrated multi-organ protection, and from reactive risk mitigation to proactive continuum-based intervention.

## Data Availability

No datasets were generated or analysed during the current study.

## Abbreviations

ACE: Angiotensin-converting enzyme
ACEi: Angiotensin-converting enzyme inhibitor
AI: Artificial intelligence
ARB: Angiotensin receptor blocker
ASCVD: Atherosclerotic cardiovascular disease
BMI: Body mass index
cAMP: Cyclic adenosine monophosphate
CI: Confidence interval
CKD: Chronic kidney disease
CKM: Cardiovascular-kidney-metabolic
CVOT(s): Cardiovascular outcomes trial(s)
DKD: Diabetic kidney disease
DPP-4: Dipeptidyl peptidase-4
eGFR: Estimated glomerular filtration rate
eNOS: Endothelial nitric oxide synthase
GIP: Glucose-dependent insulinotropic polypeptide
GLP-1: Glucagon-like peptide-1
GLP-1R: Glucagon-like peptide-1 receptor
GLP-1RA: Glucagon-like peptide-1 receptor agonist
HbA1c: Glycated hemoglobin
HF: Heart failure
HFpEF: Heart failure with preserved ejection fraction
HFrEF: Heart failure with reduced ejection fraction
HR: Hazard ratio
ICAM-1: Intercellular adhesion molecule-1
IL: Interleukin
KCCQ-CSS: Kansas City Cardiomyopathy Questionnaire Clinical Summary Score
LVEF: Left ventricular ejection fraction
MACE: Major adverse cardiovascular events
MAPK: Mitogen-activated protein kinase
MARD: Mild age-related diabetes
MASH: Metabolic dysfunction-associated steatohepatitis
MOD: Mild obesity-related diabetes
NF-κB: Nuclear factor kappa-light-chain-enhancer of activated B cells
NLRP3: NLR family pyrin domain containing 3
NNT: Number needed to treat
NO: Nitric oxide
PKA: Protein kinase A
RAAS: Renin-angiotensin-aldosterone system
RAGE: Receptor for advanced glycation end products
RCT: Randomized controlled trial
RR: Relative risk
SGLT2: Sodium-glucose cotransporter-2
SIDD: Severe insulin-deficient diabetes
SIRD: Severe insulin-resistant diabetes
T2DM: Type 2 diabetes mellitus
TGF-β: Transforming growth factor beta
TNF-α: Tumor necrosis factor alpha
UACR: Urine albumin-to-creatinine ratio
VCAM-1: Vascular cell adhesion molecule-1

## Trial acronyms

AMPLITUDE-O: Effect of Efpeglenatide on Cardiovascular Outcomes
CONFIDENCE: Finerenone Combined with Empagliflozin in Adults with CKD and Type 2 Diabetes
ELIXA: Evaluation of Lixisenatide in Acute Coronary Syndrome
EXSCEL: Exenatide Study of Cardiovascular Event Lowering
FIDELITY: Finerenone in Chronic Kidney Disease and Type 2 Diabetes: Combined FIDELIO-DKD and FIGARO-DKD Trial Programme Analysis
FIDELIO-DKD: Finerenone in Reducing Kidney Failure and Disease Progression in Diabetic Kidney Disease
FIGARO-DKD: Finerenone in Reducing Cardiovascular Mortality and Morbidity in Diabetic Kidney Disease
FLOW: Evaluate Renal Function with Semaglutide Once Weekly
FINEARTS-HF: Finerenone Trial to Investigate Efficacy and Safety Superior to Placebo in Patients with Heart Failure
HARMONY: Albiglutide and Cardiovascular Outcomes in Patients with Type 2 Diabetes
LEADER: Liraglutide Effect and Action in Diabetes: Evaluation of Cardiovascular Outcome Results
PIONEER 6: Peptide Innovation for Early Diabetes Treatment 6
RECAP: Retrospective Evaluation of Combination Antidiabetic Pharmacotherapy
REWIND: Researching Cardiovascular Events with a Weekly Incretin in Diabetes
SELECT: Semaglutide Effects on Cardiovascular Outcomes in People with Overweight or Obesity
SOUL: Semaglutide Cardiovascular Outcomes Trial
STEP-HFpEF: Semaglutide Treatment Effect in People with Obesity and HFpEF
SUMMIT: Tirzepatide for Heart Failure with Preserved Ejection Fraction and Obesity
SURPASS-CVOT: Tirzepatide vs. Dulaglutide on Major Adverse Cardiovascular Events
